# Potassium-Chloride Cotransporter 3 Interacts with Vav2 to Synchronize the Cell Volume Decrease Response with Cell Protrusion Dynamics

**DOI:** 10.1371/journal.pone.0065294

**Published:** 2013-05-28

**Authors:** Adèle Salin-Cantegrel, Masoud Shekarabi, Sarah Rasheed, François M. Charron, Janet Laganière, Rebecca Gaudet, Patrick A. Dion, Jean-Yves Lapointe, Guy A. Rouleau

**Affiliations:** 1 Centre of Excellence in Neuroscience of University of Montreal, Montréal, Québec, Canada; 2 Centre Hospitalier de l′Université de Montréal Research Centre, Montréal, Québec, Canada; 3 Department of Physics, Université de Montréal, Montréal, Québec, Canada; 4 Department of Pathology and Cell Biology, Université de Montréal, Montréal, Québec, Canada; 5 Montreal Neurological Institute, McGill University, Montréal, Québec, Canada; Aix Marseille University, France

## Abstract

Loss-of-function of the potassium-chloride cotransporter 3 (KCC3) causes hereditary motor and sensory neuropathy with agenesis of the corpus callosum (HMSN/ACC), a severe neurodegenerative disease associated with defective midline crossing of commissural axons in the brain. Conversely, KCC3 over-expression in breast, ovarian and cervical cancer is associated with enhanced tumor cell malignancy and invasiveness. We identified a highly conserved proline-rich sequence within the C-terminus of the cotransporter which when mutated leads to loss of the KCC3-dependent regulatory volume decrease (RVD) response in *Xenopus Laevis* oocytes. Using SH3 domain arrays, we found that this poly-proline motif is a binding site for SH3-domain containing proteins *in vitro*. This approach identified the guanine nucleotide exchange factor (GEF) Vav2 as a candidate partner for KCC3. KCC3/Vav2 physical interaction was confirmed using GST-pull down assays and immuno-based experiments. In cultured cervical cancer cells, KCC3 co-localized with the active form of Vav2 in swelling-induced actin-rich protruding sites and within lamellipodia of spreading and migrating cells. These data provide evidence of a molecular and functional link between the potassium-chloride co-transporters and the Rho GTPase-dependent actin remodeling machinery in RVD, cell spreading and cell protrusion dynamics, thus providing new insights into KCC3's involvement in cancer cell malignancy and in corpus callosum agenesis in HMSN/ACC.

## Introduction

Mutations in the potassium chloride cotransporter 3 (KCC3) gene cause hereditary motor and sensory neuropathy with agenesis of the corpus callosum (HMSN/ACC), a severe neurodegenerative disorder characterized by neuronal and axonal swelling [1]–[3]. C-terminal domain truncation of KCC3 and its defective transit to the plasma membrane are the major pathogenic mechanisms in HMSN/ACC that lead to the inactivation of the cotransporter, which fails to respond to swelling [1], [4]–[6]. We have determined that KCC3 truncation disrupts functional protein-protein interactions, such as KCC3 interaction with the brain-type creatine kinase (CK-B) [5], [6]. However, a co-morbid effect of KCC3 loss-of-function is the aberrant pathfinding of callosal axons, which migrate along the brain midline to form Probst's bundles instead of bridging the two brain hemispheres [3]. Conversely, gain-of-function through increased *KCC3* expression correlates with enhanced aggressiveness and invasiveness of human malignancies, including cervical, breast, ovarian cancers and astrocytomas [7]–[12].

In response to cell swelling, most potassium chloride cotransporters (KCC1, KCC3 and KCC4) are activated and cause an electroneutral ion co-transport as part of the regulatory volume decrease (RVD) response [13]. It has been previously demonstrated that KCC's regulation during RVD is dependent on cytoskeleton integrity and its dynamic modification. In particular, hypo-osmotic challenges cause dramatic re-organization of cortical actin while F-actin destabilizing agents slow down swelling recovery, providing evidence of a functional role of actin re-organization in the RVD response [14]–[17]. Actin cytoskeleton dynamics are modulated by RhoA, Rac1 and Cdc42, and require their guanine nucleotide exchange factor (GEFs)-dependent activation [18]. Among the GEFs, Vav2 has been shown to play a crucial role in RVD. Notably, Vav2 phosphorylation/activation by Src induces swelling-mediated activation of K^+^ and Cl^−^ channels and cell volume recovery [19]. In addition, Vav2 is a member of the Vav family of protooncogenes and has been involved in a large array of basic cellular processes such as cell proliferation, cell adhesion, cell spreading and cell migration as well as in neuronal specific dynamics such as neurite outgrowth, dendrite remodeling and commissural axon migration in the central nervous system [20]–[22].

Although mechanisms whereby KCC3 inactivation provides some explanations for axonal swelling in HMSN/ACC, little is known about how KCC3 loss-of-function might lead to the migration anomalies of commissural neurons in the brain. In addition, functional and physical molecular interactions linking KCC3 gain-of-function to increased malignancy and invasiveness of tumor cells are still poorly understood. In this study, we provide evidence that KCC3 interacts with the active form of Vav2. This interaction occurs preferentially within cell membrane protrusions induced by hypotonic conditions or within lamellipodia of spreading and migrating cells. These findings provide new insights into how KCC3 gain-of-function could favor cancer cell invasion while KCC3 loss-of-function might affect commissural axons crossing of the brain midline in HMSN/ACC.

## Materials and Methods

### Ethics statement

Comités d′évaluation scientifiques et d′éthique de la recherche (Centre de Recherche du CHUM); Ethical approval number No. ND04.046 (Études génétique portant sur les neuropathies).

### Constructs

A construct containing the full-length KCC3b cDNA in the pGEM vector was kindly provided by Dr. David Mount (Harvard Medical School, Boston, MA, USA). To allow the expression of KCC3 protein in mammalian cells, the full-length cDNA in pGEM was subcloned into the pcDNA 3.1C vector (Invitrogen) as described previously [4], [5]. Site-directed mutagenesis was performed using appropriate primers to produce the PGPP→PGQA exchange, which modified two conserved proline residues of the putative SH3-domain binding site and generated the KCC3^mPro^ mutant protein. Wild-type or mutated C-terminal domain of KCC3 were inserted into the pGEX 3T-1 vector (*GE Life Sciences*), which allows the production of GST-fused proteins as described previously [6].

### Flux assay

The experiments were performed as previously described [4]–[6]. Briefly, the pGEMHE templates were linearized downstream from the 3′-end of the coding sequence with *Not*I enzymatic digestion, and the cRNAs were transcribed *in vitro* using T7 RNA polymerase. *Xenopus laevis* oocytes were surgically harvested and defolliculated at room temperature for 2 h with 2 mg/ml collagenase A. Stage V and VI healthy oocytes were injected with 50 nl of water with or without cRNA (total KCC3 cRNA injected was 10 ng/oocyte). After injection, oocytes were incubated at 18°C for 5 days in Barth's solution (90 mM NaCl, 3 mM KCl, 0.82 mM MgCl_2_, 0.74 mM CaCl_2_, 10 mM HEPES/Tris, 5% (v/v) horse serum, pH 7.6) to allow for recovery and protein expression. The activity of KCC3 was determined by assessing ^86^Rb^+^ uptake in groups of 8–12 oocytes. The uptake was measured at 32°C using a standard protocol: a 60 min incubation period in a hypototonic medium (52 mM Na-Cyclamate, 3.3 mM KCl, 0.74 mM CaCl_2_, 0.82 mM MgCl_2_, 10 mM HEPES/Tris, pH 7.4) with 10 µM ouabain, was followed by an incubation in an uptake medium containing 49 mM NaCl, 15 mM Na-Cyclamate, 0.74 mM CaCl_2_, 0.82 mM MgCl_2_, 10 mM HEPES/Tris, pH 7,4, 30 mM ^86^Rb^+^-Cl^−^ and 10 µM ouabain. The KCC3-dependent uptake of ^86^Rb^+^ was deduced by exposing groups of cRNA-injected oocytes to 1 mM furosemide. The uptake experiment was stopped after 60 min by five washes in ice-cold uptake solution without the isotope, to remove extracellular fluid tracer. The oocytes were lysed in 10% sodium dodecyl sulfate and tracer activity was measured for 2 min in a liquid scintillation counter.

### SH3 domain array

The experiments were performed following the manufacturer instructions (*Panomics*). Biotin-conjugated peptides composed of the 15 a.a. sequence overlapping the proline-rich motif were synthetized to generate a wild-type KCC3 peptide (*N^term^*-LLNMPG**PP**RNPEGDE-*C^term^*) and a mutated peptide (*N^term^*-LLNMPG**QA**RNPEGDE-*C^term^*). Briefly, 15 µl of the biotinylated peptides (1.5 µg) was coupled with streptavidin-HRP in blocking solution for 30 min at 4°C. The SH3 arrays were re-hydrated/washed, blocked in the provided solutions for 2 h at room temperature and then incubated with conjugated peptide in blocking solution for 1 h. The membranes were then washed tree times and revealed using provided detection buffers before exposition to a photoreactive film.

### GST-pull down assay

The wild-type and mPro forms of the C-terminal domain of KCC3 cloned in pGEX were propagated in the bacterial strain BL21. A single colony was incubated in a 3 ml culture of LB (containing 100 µg/ml ampicilin), grown overnight at 37°C and used to produce large-scale cultures, which were grown until OD_600 nm_ = 0.6–0.8 at 30°C and incubated with 500 µM IPTG for an additional 30 min to 2 h at 30°C and a protein lysate was extracted in 0.1% PBS-triton. The expression of the fusion protein was assessed by Western blot using a GST-specific antibody. The fusion protein was purified using standard procedures on glutathione-sepharose beads (*GE Life Sciences*). HeLa cell protein lysate suspended in binding solution was first cleared for 30 min at 4°C with glutathion-sepharose beads to eliminate non-specific interaction with the beads and then incubated with the immobilized GST proteins. The binding assays were performed for 1 h at 4°C under rocking movement. Following incubation, the beads were rinsed three times for 1 h at 4°C in RIPA buffer and carefully drained. The washed beads containing the purified complexes were dissociated by boiling for 10 min in sample buffer, loaded on an SDS-PAGE gel and processed for immuno-blotting. HeLa (human cervix carcinoma) were obtained from *ATCC*. Monoclonal anti-GST primary antibody was obtained from *Clontech*. Vav2 was detected with a specific antibody (*Abcam*).

### Co-immunoprecipitation

Immunoprecipitation of Vav2 with KCC3 was achieved using the *Dyna Invitrogen beads* preparation following the manufacturer instructions. Briefly, KCC3 and KCC3^mPro^ were transiently expressed in HeLa cells, which robustly express Vav2 endogenously. The cells were then lysed in RIPA buffer (50 mM Tris-HCl pH 7.8, 150 mM 5M NaCl, 1% Triton X-100, 0.5% Sodium Deoxycholate, 0.1% SDS), centrifuged and the supernatant was transferred to the magnetic beads coupled with KCC3 or Vav2 antibody. After three successive washes the complexes were dissociated in denaturing loading buffer and process for SDS-PAGE and immuno-blot detection.

### Immunocytochemistry

HeLa cells were seeded on sterile plastic coverslips in six-well plates and transfected with 4 µg of wild-type KCC3, KCC3^mPro^ and KCC3^ΔCterm^ using lipofectamine 2000 (*Invitrogen*), according to the manufacturer's instructions. For immuno-staining the cells were incubated for 24 h in Dulbecco's modified Eagle's medium (DMEM, *GIBCO*) containing 10% fetal bovine serum (FBS), then washed in PBS and fixed in 4% paraformaldehyde in PBS at room temperature for 10 min. Fixed cells were washed in PBS before being permeabilized in PBS containing 0,2% Triton X-100 (*Sigma*) at room temperature for 10 min. Permeabilized cells were then washed in PBS, incubated for 1 h in blocking solution (10% NGS in PBS) and transferred to a 5% NGS in PBS solution containing KCC3 (*Abnova*) or Vav2 antibodies (*Abcam*) at an appropriate dilution. After an overnight incubation, coverslips were washed in PBS and were then incubated for 60 min in 5% NGS in PBS containing fluorescent secondary antibodies. The coverslips were finally washed in PBS and mounted for fluorescent or confocal microscopy.

Stable transfection of wild-type KCC3 in HeLa cells was achieved by selecting the transfected cells in G418 containing medium for several days and isolating individual KCC3 expressing clones.

### Hypotonic challenge

Isotonic conditions for HeLa cells were established at (∼320 mOsm) in a medium containing (in mM): 105 NaCl, 6 KCl, 1 MgCl2, 1.5 CaCl2, 10 glucose, 10 HEPES, and 90 mannitol (pH 7.4). Hypotonic challenge and cell swelling was achieved in a hypotonic solution (∼240 mOsm) where mannitol was omitted. The experiments were performed under standard cell culture conditions at 5% CO_2_ and 37°C for 5 min or 10 min. The effects of the isotonic conditions or hypotonicity exposure were followed using a live-stage station of microscopy imaging for 15 min.

### Scratch/wound healing assay

HeLa cells stably expressing KCC3 or not were seeded in 6 well plates, cultured in a tight monolayer (∼90% confluent) and starved in low serum media the day prior to the assay. The day of the assay, four independent simulations of wounds were made, then the cells were washed in culture medium several times to remove cell debris and allowed to migrate in complete medium (10% FBS in DMEM) at 37°C for about 36 h. The experiment was then ended when closure was observed in all four wounds of one of the conditions.

### Cell spreading assay

These experiments were carried out in 24-well culture plates previously coated with poly-lysine or not (control: ‘glass’). Forty thousand cells stably expressing KCC3 or not were transferred into the wells and allowed to attach for 1 h at 37°C. Media was then discarded, unattached cells were removed by two washes of PBS, fixed and stained with rhodamine–phalloidin for F-actin. Analysis of cell spreading was based on morphology: cells that assumed long and thin morphology were considered spread cells, whereas non-spread cells retained a round shape with uneven outline. Quantification of the spreading cells correspond to counts of spread cells in 5 observation fields in a total of 3 independent experiments.

### Statistical analysis

Statistical significance was defined as two-tailed P<0.05. The significance of the differences between oocyte groups was tested by one-way ANOVA and student's *t*-test. The results were presented as means +/−SD.

## Results

### Identification of a functional and conserved proline-rich motif in the C-terminal domain of KCC3

**Figure 1:**
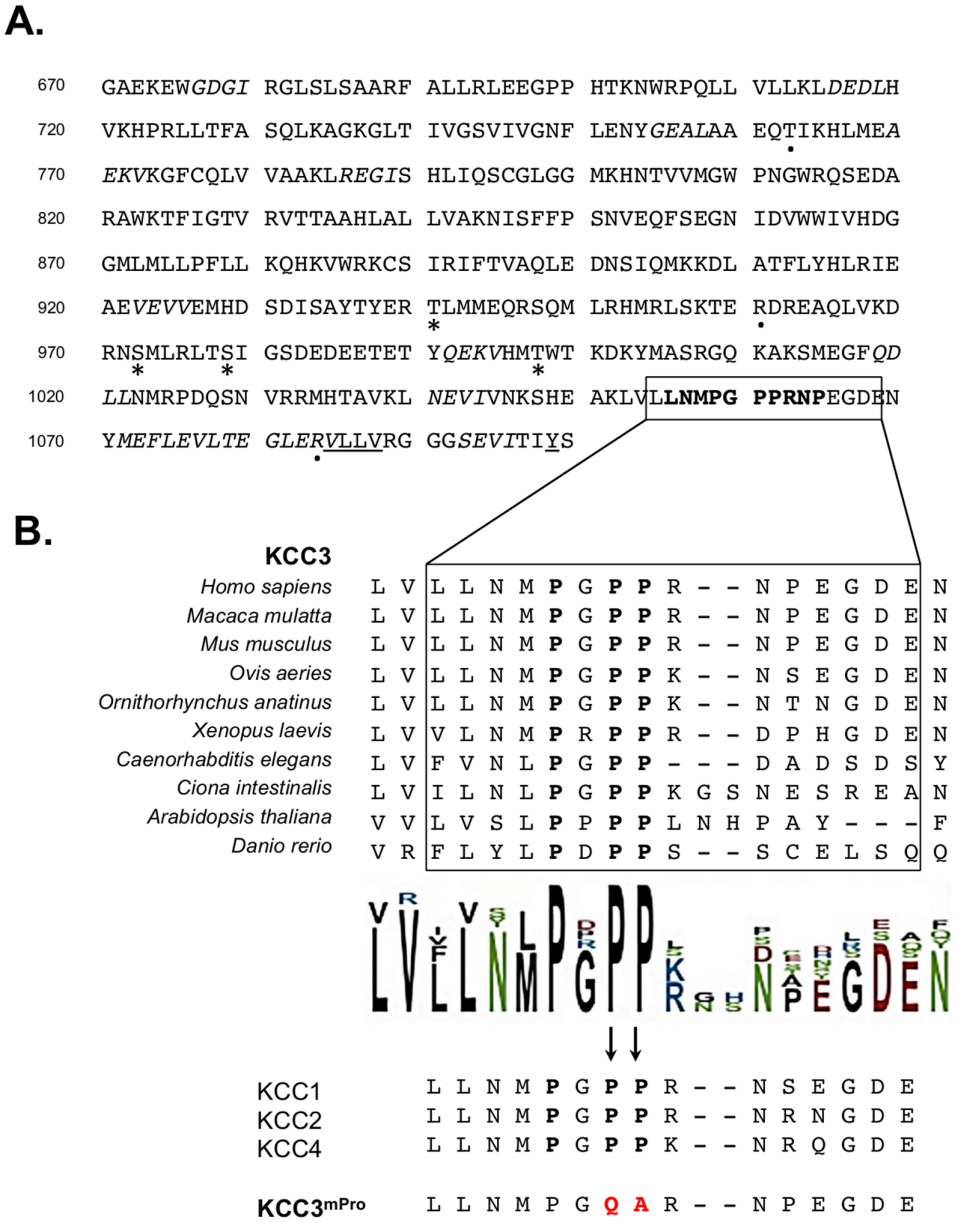
Identification of a functional proline-rich motif in the C-terminal domain of KCC3. (**A**) Protein sequence of the KCC3 C-terminal domain. The C-terminus of KCC3 contains a distal poly-proline motif indicated in bold characters which is predicted to be a binding site for SH3 domains with a non-canonical class I recognition specificity (*ELM motif data base*). The C-terminus also contains a hydrophobic tetrad and a predicted Tyrosine residue (both underlined) involved in cation-chloride co-transporter trafficking. Predicted PDZ domains interacting motifs are shown in italic. Three HMSN/ACC non-sense mutations are indicated by a dot (.). The phosphorylated sites are indicated by a star (*). (**B**) KCC3 C-terminal domain is highly conserved in the proline-rich region. The motif is conserved at 100% between KCC3 homologs but also between KCC3 orthologs KCC1, KCC2 and KCC4. The proline stretch was mutated in order to generate mPro mutant forms of KCC3 polypeptide and protein.

The C-terminus of KCC3 is a large cytosolic domain involved in interactions with functional protein partners such as the brain-type creatine kinase CK-B [5], [6]. Aside from the last 17 amino-acids of the protein that contains the interaction domain for CK-B, we identified several binding motifs *in silico*, including a non-canonical class I recognition site for SH3-domains (PXXP) (Figure 1A). This predicted binding site is a poly-proline stretch located within the last 50 a.a. of the co-transporter protein sequence and is a highly conserved motif. Notably, three proline residues are totally conserved from the plant *Arabidopsis thaliana* to humans, as well as in the KCC3 homologs (KCC1, KCC2 and KCC4). The high level of conservation suggested a role of this proline-rich sequence in KCC3 function (Figure 1B).

**Figure 2:**
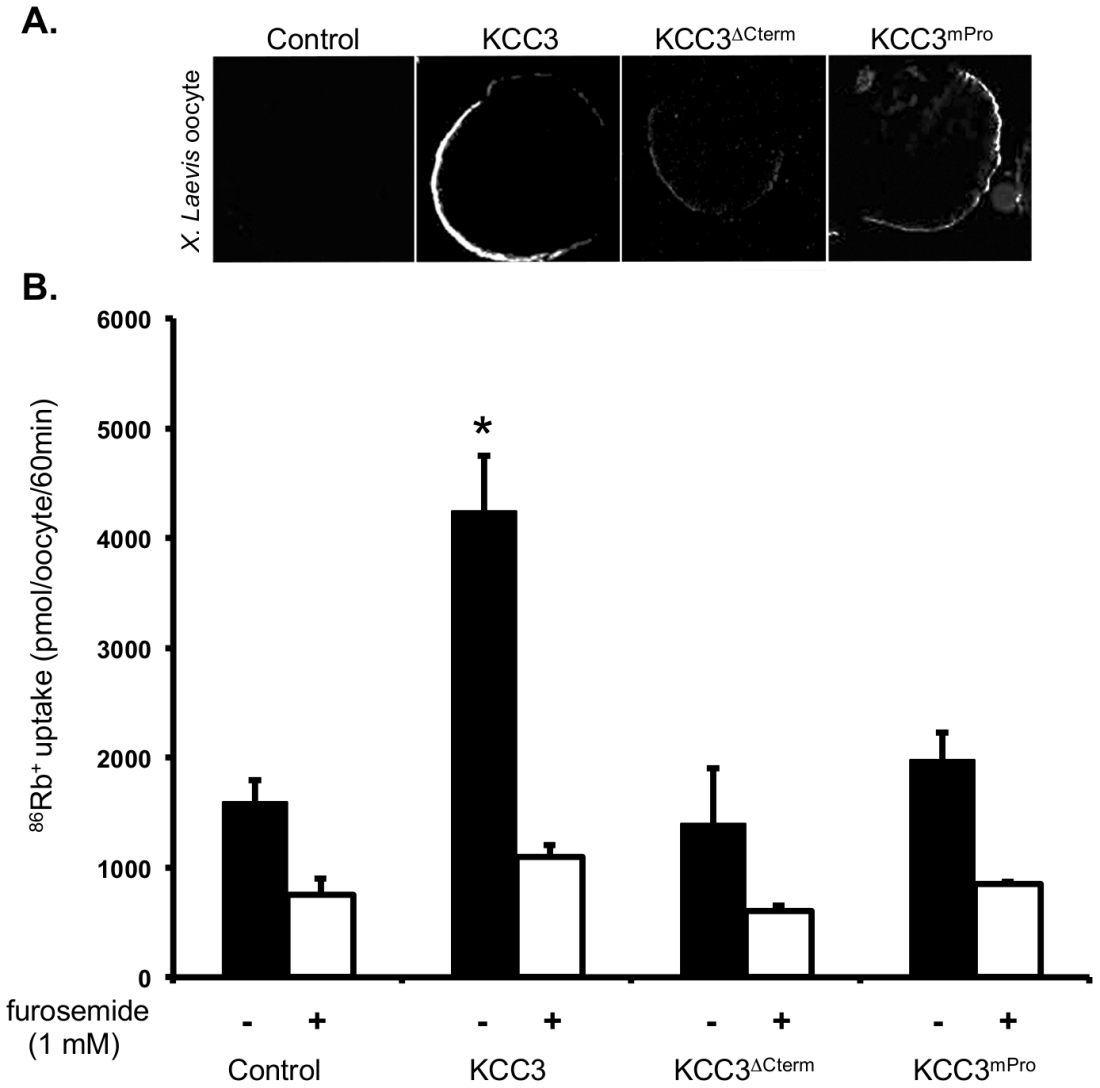
Evaluation of wild-type and mutant KCC3^ΔCterm^, KCC3^mPro^ functions in *Xenopus* oocyte flux assays. (**A**) Wild-type KCC3 and mutant KCC3^ΔCterm^, KCC3^mPro^ transit to the plasma membrane in *Xenopus Laevis* oocytes. Immunofluorescence staining of KCC3 using a specific antibody shows all generated forms at the plasma membrane of the injected oocytes. Control oocytes were injected with water instead of the wild-type and mutant KCC3 cRNAs. (**B**) Directed mutagenesis of the proline motif leads to impaired function of KCC3 in *Xenopus Laevis* oocyte flux assay. ^86^Rb^+^ flux of wild-type and mutant KCC3 is measured under hypotonic conditions in presence or absence of KCCs inhibitor, furosemide.

In order to determine whether the poly-proline site was functionally required, we used a mutant form of the cotransporter lacking the vast majority of the C-terminus (KCC3^ΔCterm^) and performed the mutagenesis of two of the proline residues in the wild type KCC3 to generate another mutant form, KCC3^mPro^ (Figure 1B). We then expressed the wild-type and mutant forms in *Xenopus Laevis* oocyte to perform ^86^Rb^+^ flux assays. However, since KCC3 mutations can affect its intracellular transit in mammalian cells [5], we first determined whether the generated forms could reach the oocyte plasma membrane. Using a specific KCC3 antibody in immuno-histochemistry experiments we found all forms at the plasma membrane, although detected at different fluorescence intensity (Figure 2A). Once assured that the co-transporters were found at the cell surface, we performed flux assays and measured KCC3 activity *via* its capacity to promote ^86^Rb^+^ fluxes. Under hypotonic conditions, exogenous expression of wild-type KCC3 in *Xenopus Laevis* oocyte resulted in a significant ^86^Rb^+^ flux (*P<0,001*; *ANOVA*), which was abolished by the application of 1 mM furosemide, a potassium-chloride co-transporter inhibitor [23], [24]. However, under these same conditions, both KCC3^ΔCterm^ and KCC3^mPro^ failed to elicit a flux similar to the wild-type forms (Figure 2B). The fact that KCC3^ΔCterm^ failed to elicit a significant flux is consistent with previous work on KCC1 and KCC3 showing reduced response of truncated forms to hypotonic conditions [1], [4], [25], [26]. This also demonstrated that KCC3^mPro^ function was impaired and that the prolines integrity was required for KCC3 activity.

### The C-terminal domain of KCC3 mediates a direct interaction with Vav2

**Figure 3:**
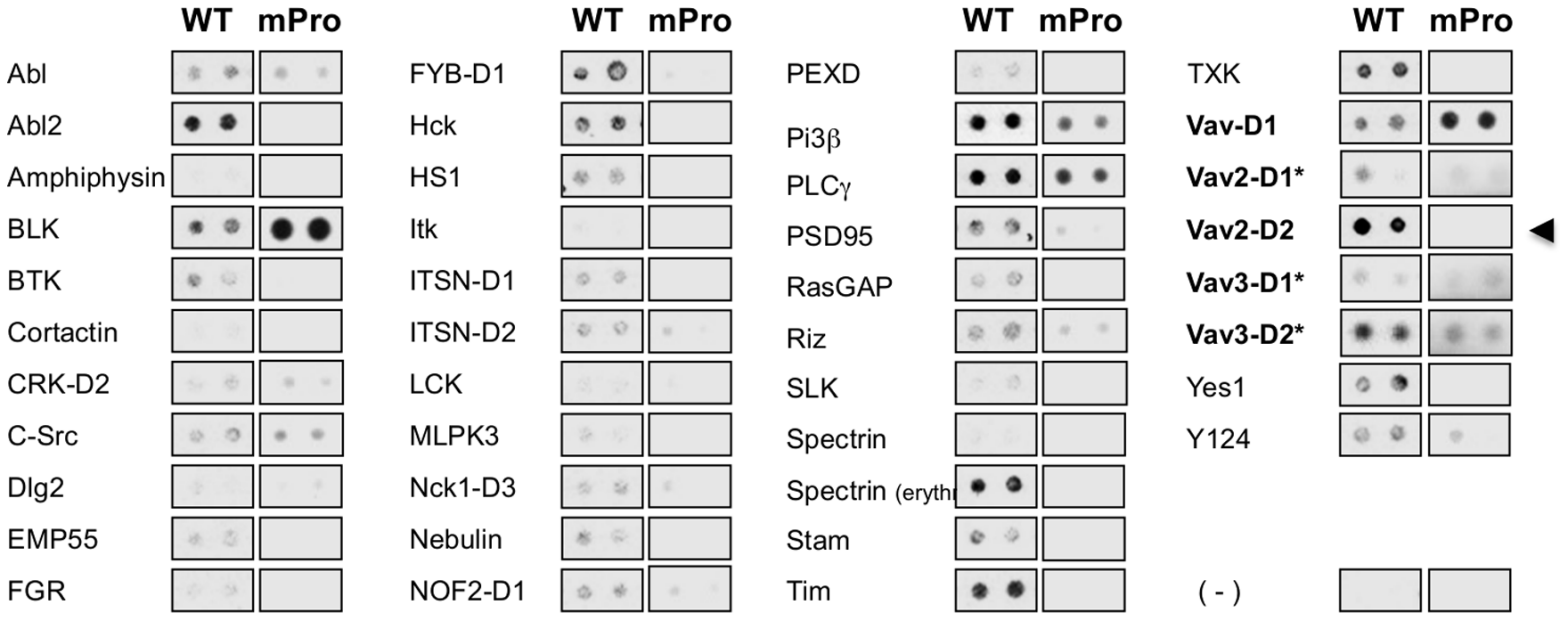
KCC3 proline-rich segment mediates binding to Vav2 SH3-domain. The proline-rich sequence interacts with a collection of SH3 domains detected after incubation of a biotinylated wild-type peptide (LLNMPG**PP**RNPEGDE) with a commercially available SH3 domain array (*Panomics*). Wild-type peptide binds to the second SH3 domain (D2) of the Vav proteins Vav2 and Vav3 but not to the SH3-domain of cortactin. To control for binding specificity of the wild-type peptide on the array, a peptide mutated for two proline residues (LLNMPG**QA**RNPEGDE) was used in parallel experiments.

Since the proline-rich motif could interact with SH3-domain containing proteins, we used commercially available SH3 domain arrays as an initial *in vitro* approach to determine if the poly-proline sequence could interact with known SH3 domains. To detect interactions, we probed the commercial SH3 domains membrane array with a wild-type peptide (WT), which contained the proline-rich sequence of interest (Figure 3). Several possible interactions with the polypeptide were detected, suggesting that the proline-rich stretch is mediating binding to SH3 domain containing partners. Notably, the second SH3-domain of Vav2 (Vav2-D2) and of its homolog Vav3 (Vav3-D2) both bound specifically with the wild-type peptide. We also detected interactions with SH3 domains of other proteins possibly involved in cell volume regulation (e.g. PLCγ and PI3β) [19]. To address issues about the specificity of all the detected interactions, we probed SH3 domain arrays with a mutated peptide (mPro) in which two proline residues were exchanged and we aimed at eliminating non-specific binding, which are still detected with the mutated peptide. Using this complementary approach, we observed retained interaction of the PLCγ and PI3β SH3 domains with the mutated peptide mPro, whereas Vav2-D2 interaction with the peptide was noticeably impaired by the amino-acid exchange. Given these observations and the established role of Vav2 in cell volume decrease, commissural axon migration and oncogenesis, we decided to focus our study on this interacting partner.

**Figure 4:**
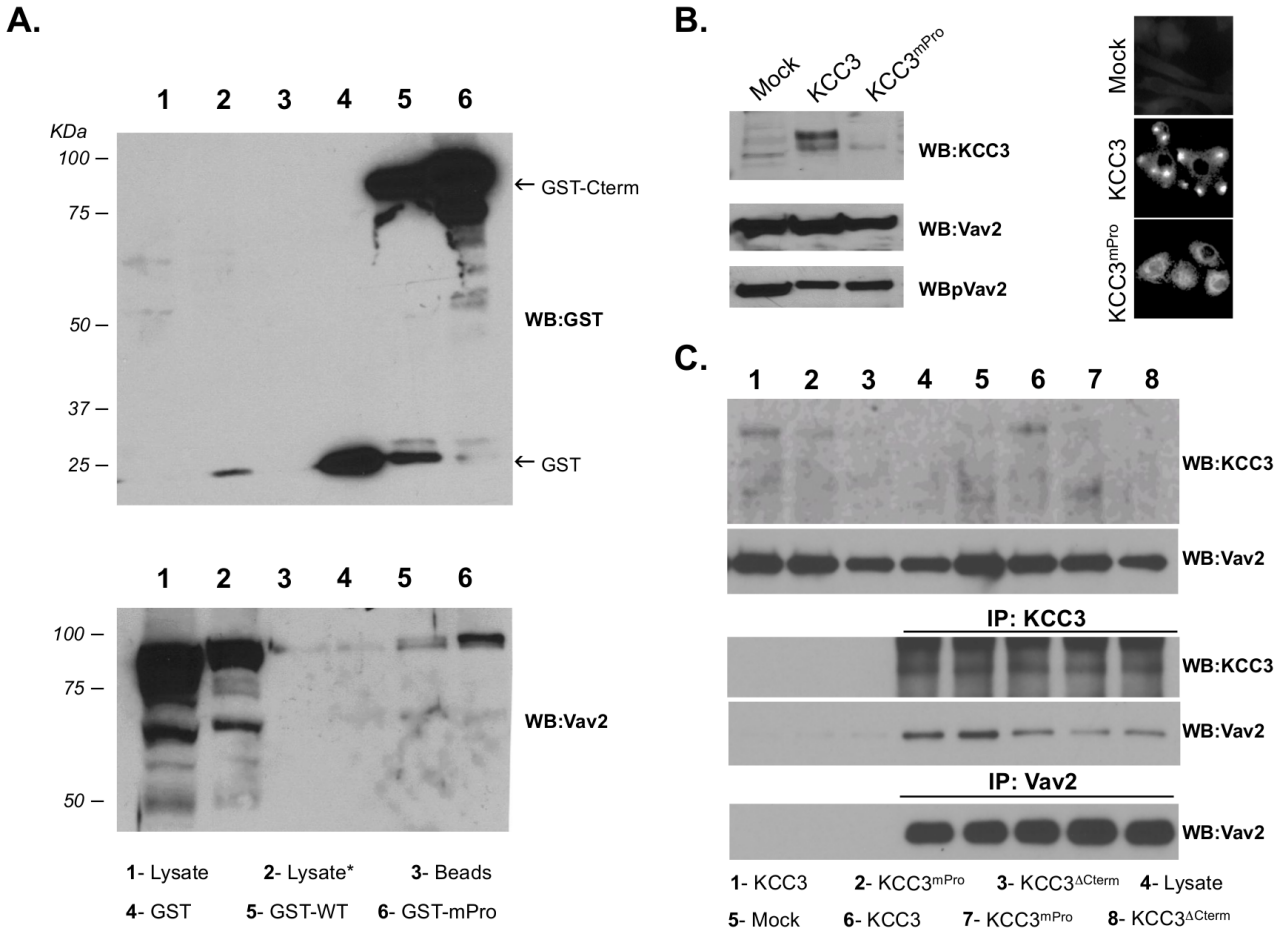
Vav2 interacts with the C-terminal domain of KCC3. (**A**) Vav2 endogenously expressed by HeLa cells binds to the C-terminal domain of KCC3 in GST-pull down assays. Lysate  =  whole protein lysate extracted from HeLa cells; Lysate*  =  pre-cleared HeLa cell protein lysate incubated with sepharose beads; GST  =  pre-cleared protein lysate incubated with GST only; GST-WT  =  pre-cleared protein lysate incubated with chimeric protein where KCC3 C-teminus was fused to GST; GST-mPro  =  pre-cleared protein lysate incubated with chimeric protein where KCC3 C-terminus was mutated for two prolines (PP→QA) and fused to GST. (**B**) KCC3 and Vav2 are endogenously expressed in HeLa cells. Transient over-expression of KCC3 in HeLa cells is associated with abundant accumulation of the wild-type protein (but not the proline mutated form) in cellular protrusions. (**C**) Vav2 and KCC3 co-immunoprecipitate together in HeLa cells. Lysate  =  whole protein lysate extracted from HeLa cells; Mock  =  protein lysate extracted from HeLa cells transfected with the control empty vector; KCC3, KCC3^mPro^ and KCC3^ΔCterm^  =  protein lysate from HeLa cells transfected with the indicated KCC3 form.

In order to confirm the interaction, we performed GST pull-down experiments. We generated fusion proteins that included the whole wild-type (WT) or mutant (mPro) C-terminus of KCC3 fused to GST. Then glutathione beads coupled to the chimeric proteins were incubated with a protein lysate extracted from HeLa cells –a human cervical cancer cell line in which Vav2 is robustly expressed. Using this approach we confirmed that Vav2 did interact specifically with the C-terminal domain of KCC3 *in vitro* (Figure 4A). However, we observed no effect of the proline motif mutagenesis on Vav2 interaction in this assay. In a complementary approach, we transiently expressed KCC3, KCC3^mPro^ and KCC3^ΔCterm^ in HeLa cells and, similarly to what was noticed previously, we detected endogenous expression of KCC3 in this cervical cancer cell line [3], as well as differences in protein levels of the wild-type and mutant forms likely due to the C-terminus involvement in the protein stability [3] (Figure 4B and 4C). We performed immuno-precipitation of KCC3 with its endogenous partners and then probed the precipitated complex using a Vav2 specific antibody. Similarly to the GST pull-down results, we observed that Vav2 co-immunoprecipitated with the wild-type form of KCC3 in this *in vitro* context (Figure 4C). However, we failed to detect significant changes in Vav2 capacity to co-immunoprecipitation with the mutated form KCC3^mPro^ or truncated form KCC3^ΔCterm^ since Vav2 likely precipitate preferentially with endogenously expressed KCC3 in HeLa cells. These combined observations suggest that Vav2 directly interacts with KCC3 C-terminal domain but that the proline residues might not be absolutely necessary for KCC3 binding to Vav2.

Under isotonic conditions and at low cell density, we observed that transient overexpression of KCC3 in HeLa cells elicited the generation of cell protrusion and intense accumulation of the cotransporter within the protruding sites. Interestingly, KCC3^mPro^ seemed to fail to generate and accumulate in similar structures (Figure 4B). These observations made us investigate whether KCC3 interaction with Vav2 could be the link to plasma membrane protrusion dynamics.

### KCC3 interacts with Vav2 in plasma membrane protrusion

**Figure 5:**
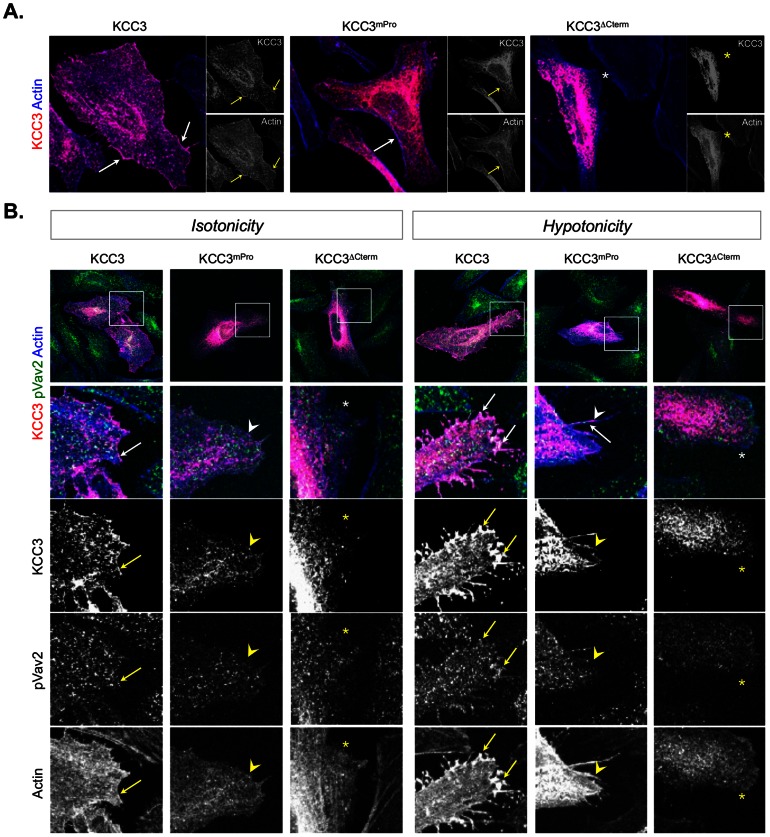
KCC3 colocalizes with the active form of Vav2 in actin-rich membrane protrusions induced by hypotonic conditions. (**A**) Distribution of wild-type and mutant KCC3 forms in transiently transfected HeLa cells. Note the aberrant distribution of the KCC3 mutant forms in the cytoplasm. (**B**) The active form of Vav2 accumulates with wild-type KCC3 but not with KCC3^mPro^ or KCC3^ΔCterm^ at the cell periphery. Wild-type KCC3 accumulates with pVav2 in actin-rich plasma membrane protrusion under hypotonic conditions (*left panel*). These results were obtained after a 10 min treatment either in an isotonic or in a hypotonic medium. In the merged images, KCC3 reactivity is indicated in red, pY-Vav2 is indicated in green and polymerized actin detected by phalloidin is indicated in blue. Arrows indicate co-localisation; the stars (*) indicate actin-rich membrane not showing KCC3 immuno-detection; the arrowheads indicate actin-rich membrane showing KCC3 immuno-reactivity but not pVav2 co-localisation.

Using confocal microscopy to assess fine protein localizations, we observed robust accumulation of wild-type KCC3 in the cell periphery, but not of KCC3^ΔCterm^ or of KCC3^mPro^ (Figure 5A). This suggests that the direct interaction of Vav2 and KCC3 might occur at the plasma membrane.

It was reported that hypotonicity challenges elicit transient formation of plasma membrane protrusions where the active/phosphorylated form of Vav2 accumulates to participate in the RVD response [17]. Under isotonic conditions, wild-type KCC3 accumulation partially co-localized with the active/phosphorylated form of Vav2 at the cell periphery (Figure 5B). However, although we knew that KCC3 was activated by hypotonic conditions (Figure 2B), we had no evidence that the plasma membrane distribution of the cotransporter could be locally altered by hypotonicity as well. Therefore, we subjected HeLa cells over-expressing KCC3 to a hypotonic challenge –which was monitored for cortical actin re-organizations– and immuno-stained for KCC3 protein using a specific antibody. In these conditions, we observed intense KCC3 accumulation in hypotonicity-induced membrane protrusions. Under these same conditions, we also evaluated whether cell swelling might impact KCC3/Vav2 interaction using a specific antibody recognizing the active/phosphorylated form of Vav2. We observed that wild-type KCC3 protein, (but not the mutant forms), accumulated within hypotonicity-induced plasma membrane protrusions and strongly co-localized with the active form of Vav2 at protruding sites located at the base of the protrusions (Figure 5B). This suggests that KCC3 and Vav2 activities collaborate at plasma membrane protruding site through a local interaction to modulate protrusion dependent processes.

### KCC3 is involved in processes dependent on actin dynamic

**Figure 6:**
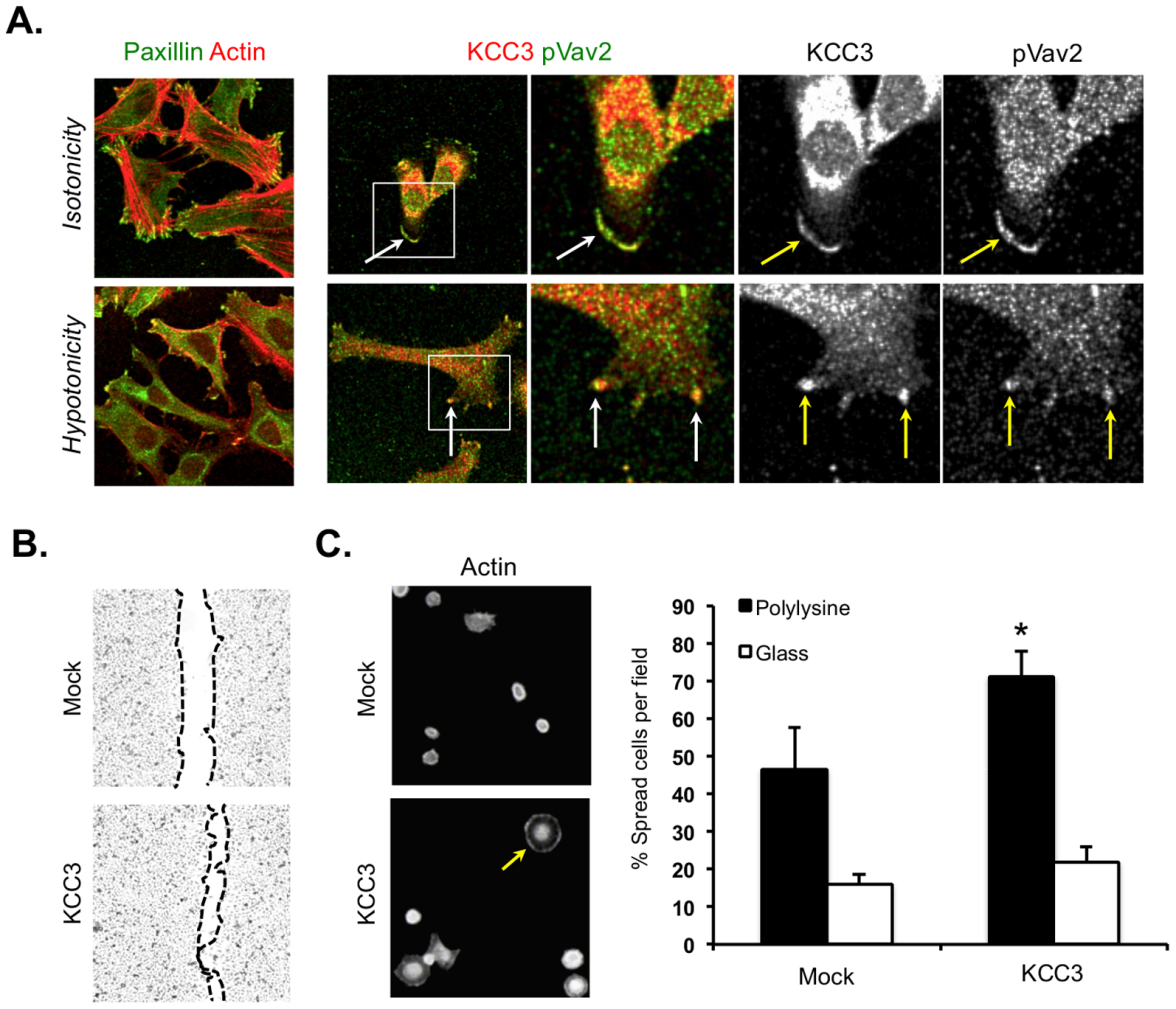
KCC3 is involved in processes dependent of actin structural dynamic. (**A**) Involvement of KCC3 in the formation of in cell protrusions. KCC3 co-localizes with the active phosphorylated form of Vav2 at lamellipodia-rich migrating front of HeLa cells that stably overexpress KCC3 (*top panel*). HeLa cells stably expressing the wild-type form of KCC3 were subjected to a hypotonic choc and show intense co-localization of KCC3 and pY-Vav2 to hypotonicity-induced membrane protrusions (*Low panel*). Involvement of KCC3 in these processes was assessed after a 5 min treatment in this experiment. On the merged image, KCC3 is indicated in red and Vav2 is stained in green. Co-localization is observed by the overlapping of the two stainings seen as a yellow signal. (**B**) KCC3 role in cell migration. Stable overexpression of KCC3 in cervical cancer (HeLa) cells accelerates wound closure in scratch assays experiments. (**C**) Spreading assay determines novel role of KCC3 in cell spreading. HeLa cells were visualized for their polymerized actin with rodamin-phalloidin. Quantification of the spreading cells on poly-lysine coated and uncoated slides (n = 3).

Vav2 participates in several processes dependent of actin dynamic including cell migration, RVD response but also cell spreading. KCCs have been involved in several of these mechanisms, namely cell volume decrease in response to swelling and cell invasion. In order to perform complementary functional assays, we generated a HeLa cell line in which KCC3 was stably overexpressed. Consistent with our observations in Figure 5, we observed that exposure of these HeLa cells to hypotonicy elicited the generation of KCC3- and Vav2-rich membrane protrusions (Figure 6A).

There were evidences of KCC3 accumulation at the leading front of migrating cells under isotonic conditions (Figure 5A, 5B and 6A) where KCC3 is known to colocalize with lamellipodia markers [5]. To further investigate aspects of KCC3 involvement in cell migration we performed *in vitro* scratch assays using HeLa cell lines that stably overexpressed or not KCC3. We observed that KCC3 stable overexpression resulted in faster closure of the wounds compared to control (Figure 6B). Theses observations strongly suggested that KCC3 function was enhanced in our engineered system and are consistent with previous finding suggesting a role of KCCs in cancer cell invasion and aggressiveness [12].

To determine whether KCC3 function could be relevant to cell spreading, a process in which Vav2 was first identified by Marignani *et al*. in 2001 [22], we performed cell spreading assays. Using this approach, we observed that HeLa cells with stable KCC3 overexpression spread significantly more on adhesive substrate compared to control cells (*P<0.05*; Figure 6C). These data provide additional evidence for KCC3 involvement in fundamental actin-dependent processes, namely cell volume decrease, cell migration and cell spreading.

## Discussion

Osmotic challenge results in dynamic changes in the actin cytoskeleton and activates electrolyte and fluid efflux to restore cell volume toward its initial resting state. We provide evidence that the GEF Vav2 binds to the C-terminal domain of the ion co-transporter KCC3 to link these processes. We found that exposure to hypotonicity and the subsequent cell volume recovery mechanism elicited KCC3/Vav2 interaction within plasma membrane protrusions. Under isotonic circumstances, this interaction between KCC3 and the active form of Vav2 occurred within lamellipodia of spreading and migrating cells. This suggested that KCC3/Vav2 interaction might participate in plasma membrane protrusion formation necessary for adequate cell spreading, cell migration and RVD response.

The proline residues seem to be important but not the only ones mediating the KCC3 and Vav2 physical interaction. Though we used three independent assays to confirm KCC3/Vav2 interaction, none of these were ideal to make a quantitative assessment of the decrease affinity. [*(1)*] The membrane bound peptides assay indicated that the interaction between the two protein partners is reduced when the proline residues are mutated, not abolished. [*(2)*] The binding efficiency of wild-type KCC3 to PLCγ and PI3β SH3 domains was observed by the signal intensity on the SH3-domain arrays; PLCγ and PI3-kinases are also mediators of the actin cytoskeleton re-organization with important roles in the cell volume decrease and cell migration [19]. However, the peptide mutated for the proline residues also interacted with both PLCγ and PI3β SH3 domains, contrary to what was observed for Vav2. We therefore put PLCγ and PI3β aside from the present study. This approach alone does not exclude a possible interaction between KCC3 and these proteins and requires further investigations. [*(3)*] In the GST pull-down and co-immunoprecipitations experiments, the high exogeneous and/or endogenous level of KCC3 inherent to these assays may hinder the detection of a reduced affinity as it would saturate the amount of endogenous Vav2 molecules present.

The Vav proteins' contribution to human malignancies and their transforming potential are due to their ability to activate the small GTPases. Rho GTPases participate in numerous processes fundamental for cancer such as cytoskeletal reorganization, adhesion, cell migration, cell polarization, cell cycle progression and cell survival. To date, there have been no reports regarding the overexpression of Vav2 in human malignancies. However, Vav2 is a critical regulator of growth factor-stimulated motility in human cancers and Vav2 signaling has been implicated in cancer cell invasion and angiogenesis of human breast cancer cells and ovarian cancer cells [27]–[29]. On the other hand, KCC3 overexpression in cervical, breast and ovarian cancer has also been associated with aggressiveness of the tumor cells but only few molecular explanations for KCC3 involvement in these aspects have been found. Similarly, KCC4, a closely related cotransporter that also harbors the proline-rich region capable of interaction with Vav2, has an important role in cancer cell invasion and metastasis as well [9]. We think that this newly identified interaction provides a possible explanation on the signaling pathways in which KCC3 and KCC4 overexpression might lead to accentuated tumor cell invasion and malignancy through the collaboration of KCCs with Vav2 in cancer cell biology.

Rho GTPases also participate in neurite extension and retraction [18]. During cortex development, the Rho GTPase Rac1 is involved in neuronal migration, neurite formation, polarization and axonal guidance. Interestingly, Rac1 regulates midline pathfinding of commissural axons during corticogenesis and its cortex-restricted invalidation results in striking agenesis of the corpus callosum in mouse [30]. Vav2, a GEF for Rac1, also promotes neurite outgrowth through its role in the repetitive activation of Rac1/Cdc42 at protruding sites [31]–[34]. In addition, Vav2 regulates midline crossing of longitudinal axons during drosophila embryogenesis by acting downstream of guidance receptors [21]. During the midline crossing decision in corpus callosum development, ephrin signaling through Eph receptor tyrosine kinases promote attraction or repulsion of axonal growth cones. Interestingly, this response to ephrins is dependent of Vav2 [35]. A hallmark of the KCC3 defect in HMSN/ACC is the agenesis of the corpus callosum for which no explanation has been found so far. Agenesis of the corpus callosum in HMSN/ACC could be due to premature collapse of KCC3^−/−^ growth cones, which would affect commissural axon migration and crossing of the midline. However, partial or no agenesis of the corpus callosum has also been reported in HMSN/ACC, which suggests that the migration process is not fully abolished but only impaired. Another possibility is that callosal axons might be confused and follow an aberrant path along the brain hemisphere. In this alternative hypothesis defective KCC3^−/−^ growth cones would fail to respond properly to guidance cues (maybe ephrins), who's signaling is fundamental during corpus callosum formation. In conclusion, this newly identified interaction between KCC3 and Vav2 provides new insights on how KCC3 truncation and its loss-of-interaction with Vav2 might affect response to guidance cues and/or axonal growth and therefore lead to agenesis of the corpus callosum in HMSN/ACC.
